# The Not-So-Merry-Go-Round: Traumatic Inferior-Anterior Hip Dislocation in a 9-Year-Old

**DOI:** 10.7759/cureus.28566

**Published:** 2022-08-29

**Authors:** Lyndon Y.H. Low, MN Baig, Odhran Murray

**Affiliations:** 1 Trauma and Orthopaedics, University Hospital Galway, Galway, IRL; 2 Orthopaedics, University Hospital Galway, Galway, IRL

**Keywords:** inferior-anterior, traumatic, merry-go-round, hip dislocation, paediatric

## Abstract

Merry-go-rounds are not as innocuous as they may seem. Pediatric hip anterior-inferior dislocations are very rare and can be associated with low-energy trauma. Prompt recognition of pediatric hip dislocations is vital, and this should be treated as a time-sensitive orthopedic emergency. Closed reduction within 6 hours minimizes the risk of avascular necrosis (AVN). We present a case of a 9-year-old boy with an inferior-anterior hip dislocation following low energy trauma while playing on a merry-go-round. The patient was emergently brought to the theatre for closed reduction under general anesthesia within 6 hours. At his 12-month follow-up, he has a full range of motion without any pain.

## Introduction

In the pediatric cohort, traumatic hip dislocations constitute approximately 5% of joint dislocations. Posterior dislocations are the most common, accounting for 90% of pediatric traumatic hip dislocations. The remainder is anterior dislocations, which can be either superior-anterior (pubic) or inferior-anterior (obturator) [1,2]. Nevertheless, these are rare childhood injuries, with less than five cases of the latter reported in the literature [3]. Prompt recognition and reduction of pediatric hip dislocations are crucial to minimizing the risk of osteonecrosis [4]. This case report highlights pediatric traumatic hip dislocations, their management strategies, and potential complications.

## Case presentation

**Figure 1:**
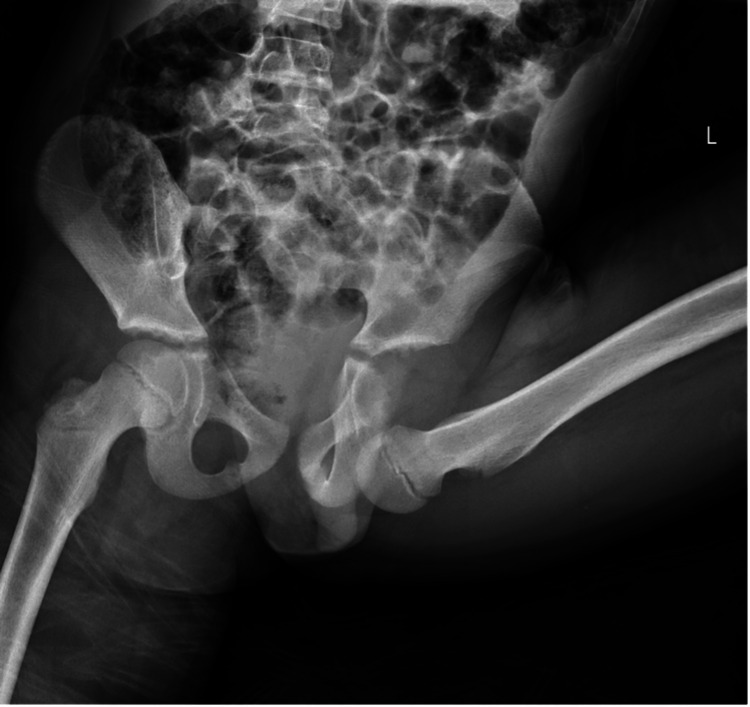
X-ray of the child demonstrating an anterior obturator type dislocation of the left hip.

We describe a case of a 9-year-old boy who presented to the emergency department with severe left hip and groin pain following a playground accident. The patient was speeding up a merry-go-round by running around it while holding on to its bars. His left foot was subsequently caught by his brother’s legs, resulting in a traction injury to his left lower limb. Radiographic investigation of his pelvis revealed an inferomedial dislocation of the left hip (Figure 1).

The patient is a fit and healthy child with no medical or surgical background. Clinically, he presented with an abducted, externally rotated, and flexed hip joint. A clinical limb length discrepancy was noted. The injury is closed, and its overlying skin is intact. A thorough neurovascular examination in the emergency department revealed strong dorsal pedis and posterior tibial pulses.

In contrast, both divisions of the sciatic nerve, i.e., common peroneal and tibial nerves, were also intact. IV fluids and analgesia were commenced, and informed consent was obtained for a closed reduction in theatre from the patient’s parents. A pre-operative computed tomography (CT) scan was decided against, given the position of the limb and its potential delay to the theatre. 

**Figure 2:**
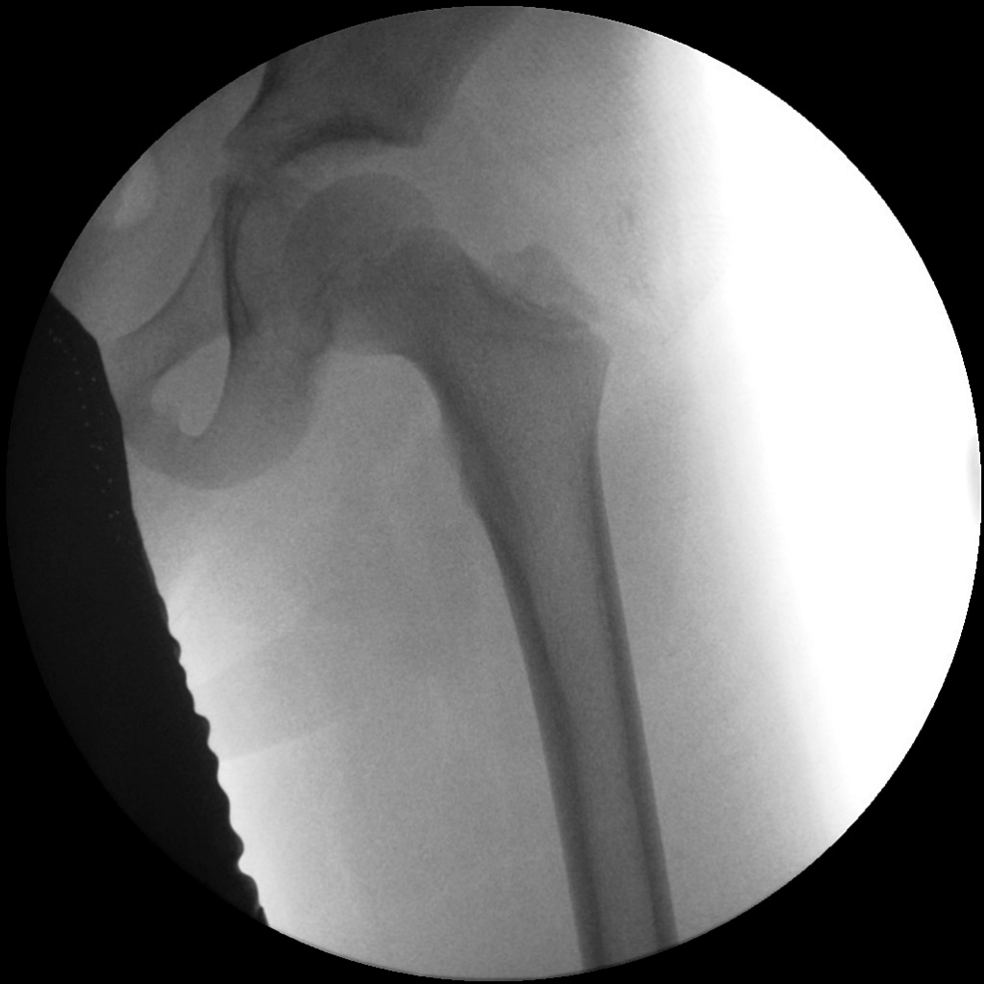
Post reduction X-ray confirming the concentric reduction of the left hip.

**Figure 3:**
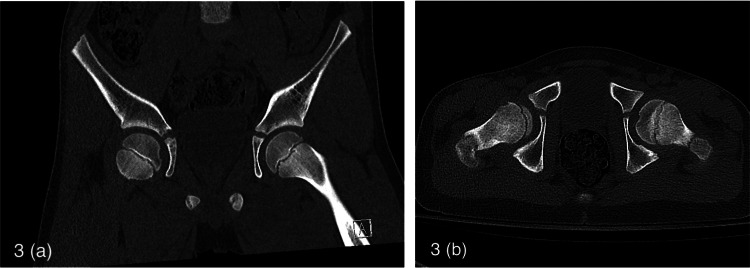
Coronal (a) and axial (b) slices of post-reduction CT scan demonstrating the position of the left femoral head in the acetabulum. No fractures or incarcerated osteochondral fragments were seen.

The patient was emergently brought to the theatre for closed reduction under general anesthesia approximately 4 hours from the presentation time. The Reverse Bigelow maneuver was performed on the theatre table, with longitudinal traction, adduction, and internal rotation to the hip, followed by extension [2,5]. The hip joint was reduced successfully on the first attempt and was confirmed with post-reduction fluoroscopy (Figure 2). The stability of the hip was also tested prior to the weaning of general anesthesia. Following that, a CT of the left hip was performed to rule out any occult fractures involving the proximal femur and/or the acetabulum to ensure that no intra-articular incarcerated fragments were found (Figures 3a, 3b). During his inpatient stay, physiotherapy was commenced to allow for toe-touch weight-bearing in an extension knee brace. This weight-bearing status was continued for a further six weeks.

**Figure 4:**
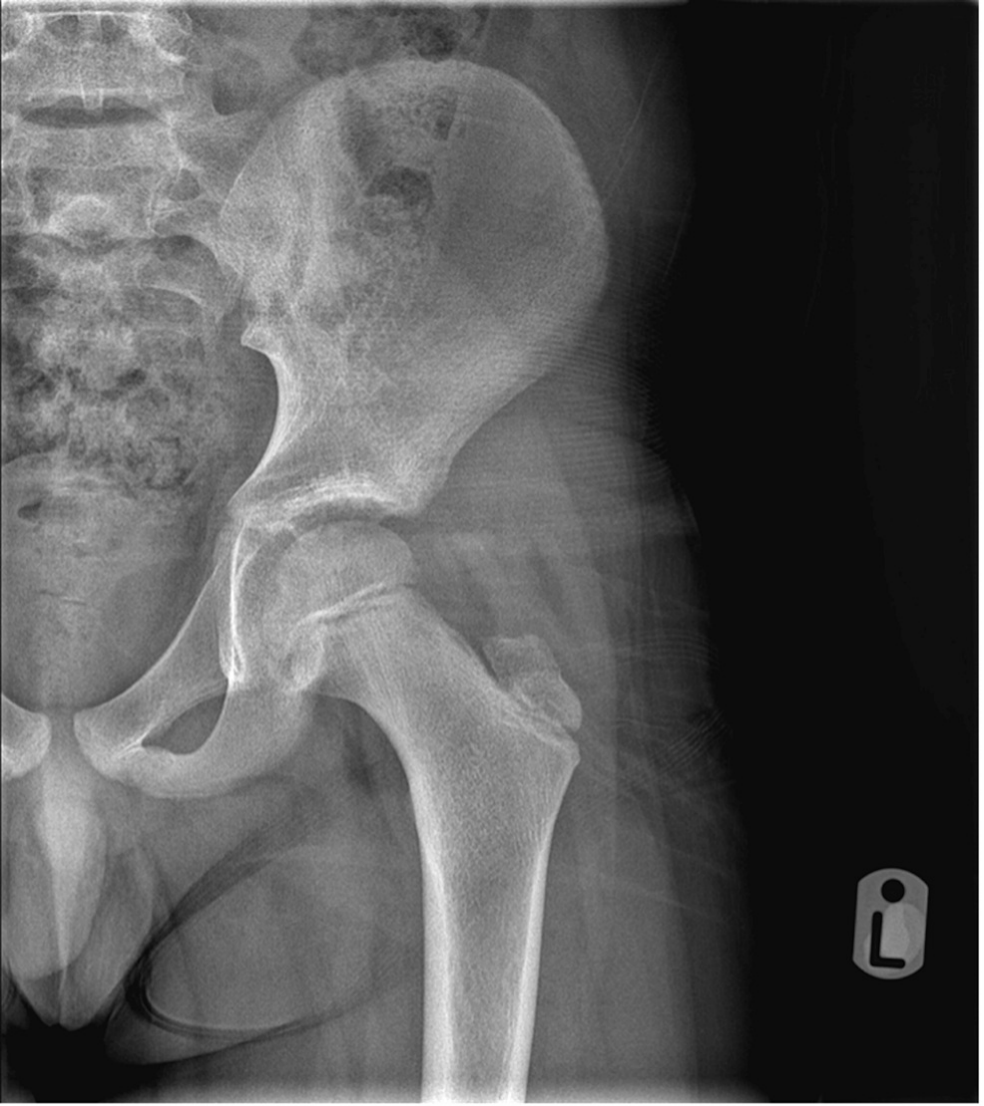
Follow-up X-Ray at 12 months without any radiological evidence of AVN.

At his 12-month follow-up, he had a full range of motion in his hip without any pain and has been actively engaging in sports at school. A normal hip X-Ray was demonstrated (Figure 4), and magnetic resonance imaging (MRI) was not performed due to a low clinical suspicion of avascular necrosis.

## Discussion

Although the hip is a relatively stable joint with static and dynamic stabilizers, traumatic hip dislocations constitute approximately 5% of pediatric joint dislocations [1]. The force required for a dislocation to occur increases with skeletal maturity, which is secondary to the relative laxity of periacetabular structures in the pediatric population. Therefore, it is thought that minor trauma can produce a dislocation below 10 years of age, whereas higher energy trauma is likely to cause dislocation in children above 12 years [4]. Posterior dislocations are the most common, accounting for 90% of pediatric traumatic hip dislocations. The remainder is anterior dislocations, which can be either superior-anterior (pubic) or inferior-anterior (obturator) [1,2]. Posterior dislocations are clinically identified by a shortened, adducted, flexed, and internally rotated lower limb. Anterior dislocations present with abducted and externally rotated limbs; however, the superior-anterior (pubic) type will be in extension, whereas the inferior-anterior (obturator) type will be in flexion [2]. Nevertheless, an abnormal position limb should raise the suspicion of hip dislocation. 

In terms of initial management specific to the dislocation, a neurovascular examination should be performed and documented to identify any concomitant injuries. Pre-reduction plain film radiographs should also be performed to guide the reduction and identify associated fractures. Promptly closed reduction within 6 hours under sedation in the emergency department or with general anesthesia in theatre is recommended [4]. It is important to note that the concentric reduction of a dislocated hip may be hindered by physeal injury, acetabular fracture, femoral head displacement, massive hematoma, and interposition of a capsular, labral, or osteochondral fragment. After a maximum of two unsuccessful gentle reduction attempts, an open reduction will need to be considered for anatomic reduction of the joint. Anterior dislocations should be approached anteriorly and vice versa to preserve residual vascularity to the femoral head, besides allowing access to repair bony or capsular defects in the direction of the dislocation [6,7]

Post-reduction X-rays should be taken to confirm concentric reduction and joint congruity. In addition, post-reduction CT or MRI provides better delineation of fragmented osseocartilaginous structures that are not readily apparent on X-ray [8]. There is no clear evidence for various post-reduction weight-bearing regimes [8]. In younger children, spica cast immobilization or bed rest with abduction splinting for up to four weeks is generally preferred, while older children and adolescents can be managed with protected weight bearing for 6 to 12 weeks to allow for the soft tissue and synovial irritation to heal [4,9]. Follow-up X-rays should be performed until skeletal maturity is reached [8].

The most common complication following hip dislocation is AVN of the femoral head, occurring in 3% to 15% of cases [4,8,10]. In the presence of a fracture-separation of the proximal femoral epiphysis (femoral head displacement), the risk of AVN increases to almost 100% [4]. Mehlman et al. demonstrated in a long-term follow-up of 42 pediatric hip dislocations that the most critical risk factor for avascular necrosis (AVN) of the femoral head was the amount of time spent dislocated. The risk of AVN was 20 times higher in those whose reduction was delayed beyond 6 hours, hence the 6-hour reduction recommendation [10].

The sciatic and superior gluteal nerve injury at the dislocation has an incidence of around 5%. The peroneal branch of the sciatic nerve is most implicated. Observation of nerve recovery and prompt reduction is recommended. Open exploration of the sciatic nerve is not warranted in most cases [4].

In adult hip dislocations, post-traumatic arthritis is a common complication. However, there is insufficient literature on adequate follow-up in pediatric hip dislocations [8]. It is, however, infrequently reported in the absence of AVN. On the other hand, it has been reported in up to 20% of patients complicated by AVN. Less commonly are recurrent hip dislocations in younger children due to the posterior acetabular cartilage pliability. This can be addressed via an open capsular repair in those with persistent instability and a radiologically confirmed tear [4].

## Conclusions

Merry-go-rounds are not as innocuous as they may seem. Pediatric hip inferior-anterior dislocations are very rare and can be associated with very low energy trauma. The take-home message is to recognize pediatric hip dislocations promptly and to treat this as a time-sensitive orthopedic emergency. Closed reduction should be attempted within 6 hours to minimize the risk of AVN.

## References

[1] Bakan ÖM, Dastan AE, Yagmuroglu K, Aktuglu K (2021). Surgical treatment of a traumatic open anterior hip dislocation in a child: a case report and review of 13 cases in the literature. Trauma Case Rep.

[2] Dawson-Amoah K, Raszewski J, Duplantier N, Waddell BS (2018). Dislocation of the hip: a review of types, causes, and treatment. Ochsner J.

[3] Ahmad S, Devkota P, Mamman KG (2015). Traumatic anterior dislocation of hip in a child - case report. Malays Orthop J.

[4] Herrera-Soto JA, Price CT (2009). Traumatic hip dislocations in children and adolescents: pitfalls and complications. J Am Acad Orthop Surg.

[5] Waddell BS, Mohamed S, Glomset JT, Meyer MS (2016). A detailed review of hip reduction maneuvers: a focus on physician safety and introduction of the Waddell technique. Orthop Rev (Pavia).

[6] Başaran SH, Bilgili MG, Erçin E, Bayrak A, Öneş HN, Avkan MC (2014). Treatment and results in pediatric traumatic hip dislocation: case series and review of the literature. Ulus Travma Acil Cerrahi Derg.

[7] Vialle R, Odent T, Pannier S, Pauthier F, Laumonier F, Glorion C (2005). Traumatic hip dislocation in childhood. J Pediatr Orthop.

[8] Furuya H, Shimamura Y, Kaneko K, Sakuramoto H, Hirata K, Arai Y (2014). Traumatic dislocation of the hip in a child caused by trivial force for age. Case Rep Orthop.

[9] Funk FJJ (1962). Traumatic dislocation of the hip in children: factors influencing prognosis and treatment. J Bone Joint Surg.

[10] Mehlman CT, Hubbard GW, Crawford AH, Roy DR, Wall EJ (2000). Traumatic hip dislocation in children. long-term followup of 42 patients. Clin Orthop Relat Res.

